# Analysis of predictors for postoperative complications after pancreatectomy––what is new after establishing the definition of postpancreatectomy acute pancreatitis (PPAP)?

**DOI:** 10.1007/s00423-023-02814-7

**Published:** 2023-02-06

**Authors:** O. Radulova-Mauersberger, F. Oehme, L. Missel, C. Kahlert, T. Welsch, J. Weitz, Marius Distler

**Affiliations:** 1 Department of Visceral, Thoracic and Vascular Surgery, University Hospital Carl Gustav Carus, Technische Universität Dresden, Fetscherstrass 74, 01307 Dresden, Germany; 2 National Center for Tumor Diseases (NCT/UCC), Dresden, Germany; 3 German Cancer Research Center (DKFZ), Heidelberg, Germany; 4 Faculty of Medicine and University Hospital Carl Gustav Carus, Technische Universität Dresden, Dresden, Germany; 5 Helmholtz-Zentrum Dresden - Rossendorf (HZDR), Dresden, Germany

**Keywords:** Pancreaticoduodenectomy, Pancreatic fistula, Postpancreatectomy pancreatitis, Postoperative hyperamylasemia, Rescue pancreatectomy

## Abstract

**Purpose:**

We aimed to analyze the predictive value of hyperamylasemia after pancreatectomy for morbidity and for the decision to perform rescue completion pancreatectomy (CP) in a retrospective cohort study.

**Methods:**

Data were extracted from a retrospective clinical database. Postoperative hyperamylasemia (POH) and postoperative hyperlipasemia (POHL) were defined by values greater than those accepted as the upper limit at our institution on postoperative day 1 (POD1). The endpoints of the study were the association of POH with postoperative morbidity and the possible predictors for postpancreatectomy acute pancreatitis (PPAP) and severe complications such as the necessity for rescue CP.

**Results:**

We analyzed 437 patients who underwent pancreaticoduodenectomy over a period of 7 years. Among them, 219 (52.3%) patients had POH and 200 (47.7%) had normal postoperative amylase (non-POH) levels. A soft pancreatic texture (odds ratio [OR] 3.86) and POH on POD1 (OR 8.2) were independent predictors of postoperative pancreatic fistula (POPF), and POH on POD1 (OR 6.38) was an independent predictor of rescue CP. The clinically relevant POPF (49.5% vs. 11.4%, *p* < 0.001), intraabdominal abscess (38.3% vs. 15.3%, *p* < 0.001), postoperative hemorrhage (22.8% vs. 5.1%, *p* < 0.001), major complications (Clavien-Dindo classification > 2) (52.5% vs. 25.6%, *p* < 0.001), and CP (13% vs. 1.8%, *p* < 0.001) occurred significantly more often in the POH group than in the non-POH group.

**Conclusion:**

Although POH on POD1 occurs frequently, in addition to other risk factors, it has a predictive value for the development of postoperative morbidity associated with PPAP and CP.

**Supplementary Information:**

The online version contains supplementary material available at 10.1007/s00423-023-02814-7.

## Introduction

Pancreatic surgery is considered a safe procedure in high-volume centers; however, infectious complications may still affect the outcome and prolong the hospital stay of patients [1–3]. A postoperative pancreatic fistula (POPF) is the most predominant and adverse complication of pancreatic surgery and occurs postoperatively in 10%–34% of patients [4, 5]. The incidence of clinically relevant POPF (CR-POPF) is 17%, and the mortality is approximately 1% [4, 6]. Aside from shifting pancreatic surgeries to high-volume centers under high-volume surgeons and improving surgical techniques, diverse perioperative conditions have been investigated in recent years to develop mitigation strategies aimed at reducing postoperative morbidity for these patients.

To enable systematic research and improve the treatment outcome, the International Study Group of Pancreatic Surgery (ISGPS) established definitions and grading for the most common postoperative morbidities related to pancreatic surgeries. The POPF classification was accepted and implemented in clinical practice worldwide and was revised and refined 11 years later [7, 8]. Various studies have been conducted to clarify the etiology and development of POPF and its complications that may trigger life-threatening hemorrhage or sepsis with organ failure. Recently, research has focused on postpancreatectomy acute pancreatitis (PPAP) as an infectious process responsible for triggering consecutive septic complications. Following the definition of acute pancreatitis based on the revised Atlanta classification, the changes in pancreatic enzyme levels that are observed postpancreatectomy and their association with complications were examined, mostly retrospectively [9–12]. Due to the lack of universal definition, various other criteria, such as radiological findings or additional biochemical parameters, were used to define postoperative pancreatitis; further, the time of occurrence differed widely in almost all studies. The ISGPS provided a universal definition and grading system for PPAP in November 2021 wherein postoperative hyperamylasemia (POH) is a part of the PPAP’s criteria, though similar to biochemical leakage in case of POPF, it was not considered clinically relevant [13]. However, the association of POH with other biochemical changes and risk factors needs further elucidation to mitigate complications.

We evaluated data from patients who underwent pancreaticoduodenectomy (PD) in our institution over a period of 7 years. In addition to the overall morbidity when POH occurred, which was our primary objective, we focused on the biochemical changes in cases where rescue complete pancreatectomy (CP) was performed due to severe septic complications. Since these cases present with complications following severe pancreatitis, we aimed to analyze their biochemical constellation and dynamics to evaluate the important predictors of prognosis. Finally, we aimed to pave the way for further prospective studies of PPAP.

## Methods

The article was written in accordance with the STROBE statement (18313558) [14]. The study protocol was approved by the local ethics committee of the Technische Universität (TU) Dresden (decision number BO-EK-542112021). Data from all patients who underwent PD as pylorus-preserving pancreaticoduodenectomy (PPPD) or classic Whipple PD from 2012 to 2019 were retrospectively collected and analyzed in an electronic database for a cohort study. All procedures were conducted at the Department of Visceral, Thoracic and Vascular Surgery of the University Hospital Carl Gustav Carus, Dresden. Demographic data, operative details, and perioperative data, including blood results (amylase, lipase, leukocytes, and C-reactive protein [CRP]) on postoperative days (PODs) 1, 3, and 5, were collected. Preoperative comorbidity was assessed according to the America Society of Anesthesiologists (ASA) classification. POH and POHL were defined when values were greater than the institution upper limit (< 0.88 μmol/(s*L) and < 1.00 μmol/ (s*L), respectively). During the studied period, no somatostatin analogs were given as a standard perioperative treatment. All patients received antibiotics cefuroxime, a second-generation cephalosporin, and metronidazole during anesthesia induction. In case of suspected or known allergy, clindamycin was administered. The reconstruction after resection was always performed as an invaginating single suture end-to-side pancreatojejunostomy. In our institution, we use PDS II (polydioxanone) 5–0 JRB-1 for the pancreatojejunostomy, and when knotting, we tie down on the intestinal wall using it as a stable suture layer to avoid damage of the pancreatic tissue. The JRB-1 needle has semicircle shape and a length of 18 mm. The thickness of the cord is 5–0 USP (United States Pharamcopoe), corresponding to 0.100–0.149 mm.

Drainage management was carried out according to the clinical standards and the fistula risk score (FRS) [15]. Patients with low (1–2) FRS did not receive any drainage. Patients with an intermediate (3–6) or high-risk (7–10) FRS were drained intraoperatively with two easy-flow lines. Levels of amylase in the drainage fluid were measured according to the clinic SOP (standard operating procedures) at POD1 and POD3. If no POPF is detected, the drainage was removed if the output was less than 800 ml. If pancreatic fistula was evident, the drainage was left and amylase was monitored every 2–3 days. Serum amylase was not included in drainage management in the study period.

Postoperative morbidity was recorded according to the Clavien-Dindo classification system (CDC) and was defined as any complication occurring after the index surgery during the length of hospital stay [16]. The ISGPS definitions and grading for POPF and postoperative hemorrhage (PPH) were used to record the postoperative data [8, 17]. Amylase and lipase activity in the drainage fluid, if a drainage tube was placed, was measured routinely on PODs 1 and 3 to detect a POPF. Radiological evidence of postoperative fluid collections in the abdominal cavity and any subsequent interventional therapy were documented. According to our SOP, if CRP is > 140 mg/l, then further diagnostic is performed. Patients receive a CT scan and if a fluid collection is present a CT-assisted drainage and resistogram-based antibiotics, according to the microbial spectrum of the intraoperative bile smear test collected.

Wound infections were documented if they were clinically relevant and led to a prolongation of the hospital stay or recurrent surgical revisions. Sepsis was defined according to the international consensus and definition [18].

Postoperative CP was performed depending on the clinical findings at a wide variable interval to the primary surgery. We aimed to perform prompt treatment and prevent further complications due to compromised intraoperative situs caused by adhesions and pancreatitis. Indication for CP was provided in the case of severe septic postoperative course with an increase of the inflammatory parameters, clinical signs of sepsis with one- or multi-organ failure, if imaging has demonstrated a severe necrotizing pancreatitis, and in case of erosion hemorrhage due to pancreatitis.

PPAP is now defined by the ISGPS as an inflammatory condition of the pancreatic stump with POH levels above an institution’s upper limit, supported by radiological and clinical findings. The latter is not specified but is summarized as a deviation from the regular postoperative course [13]. Morphological changes in the pancreatic remnants may be initially unrecognized and even difficult to distinguish from the postoperative changes. Evidence for PPAP according to the new definition is lacking because it has only recently been provided; however, most of the studies showed an association between POH and the enhanced postoperative morbidity [10, 11, 19, 20].

The primary endpoints of the study was the postoperative morbidity in both groups (patients with POH and without POH [non-POH]) and associated predictors for POPF or PPAP in cases requiring rescue CP. The secondary endpoint was the morbidity associated with POH.

### Statistical analysis

Data were analyzed using the SPSS version 21 software (IBM Corp., Armonk, NY, USA). Normality of continuous data was tested by means of the Kolmogorov–Smirnov test and through examining the frequency distributions. The homogeneity of variances was analyzed using Levene’s test. Competitive analysis was performed to compare baseline characteristics between the groups of patients categorized according to POH using the chi-square test or Fisher’s exact test for categorical variables. Unifactorial analysis of variance, Student’s *t*-test, or the Mann–Whitney *U* test was performed on continuous variables where appropriate, and the results are represented as median and interquartile range (IQR).

Logistic regression analysis was performed to determine the relationship between risk factors for POPF and rescue CP. Other variables included in the multivariate regression analysis were comorbidities, pancreatic texture, pancreatic duct diameter, and POH. All clinically relevant variables and those with a *p*-value of < 0.3 in univariate regression analysis were included in a multivariate stepwise regression model.

A *p*-value of < 0.05 was considered the threshold of statistical significance for all analyses. During the analyses, missing data were treated as missing completely at random. Thus, a complete case analysis was performed, and few patients were excluded from the analysis*.*

## Results

### Incidence of POH and association with postoperative morbidity and mortality

Data from 437 patients (57% male and 43% female) were collected and analyzed during the hospital stay. The mean patient age was 67 (IQR: 60–75) years, and the median body mass index (BMI) was 25 (IQR 22.5–28.1) kg/m^2^. Based on the ASA, the patients primarily belonged to the preoperative comorbidity grades of 2 and 3 (39.7% and 54.8%, respectively). Table 1 depicts the basic patient characteristics. We analyzed 437 patients in our database. Data for POH on POD1 were not complete for 18 patients, and all results were shown transparently in the tables. The study population was divided into two groups depending on the postoperative amylase level. Notably, the patient number for POH (*n* = 219 [52.3%]) and non-POH (*n* = 200 [47.7%]) was almost equal. Distal pancreatic resections and drainage procedures were excluded from the study. The number of cases during the study period determined the sample size.

**Table 1:** Baseline patient characteristics

	Overall	∅ Amylasemia postoperative	Amylasemia postoperative	*p*-value
Patients [*n* (%)]	419	219 (52.3)	200 (47.7)	
Sex [*n* (%)]				
m	239 (57)	122 (55.7)	117 (58.5)	0.56
w	180 (43)	97 (44.3)	83 (41.5)	
Median age [years] (IQR)	67 (60–75)	68 (62–76)	67 (58–75)	0.37
Median BMI [kg/m^2^] (IQR)	25 (22.5–28.1)	24.6 (22.2–27.2)	25.5 (23.128.4)	< 0.01
Preoperative amylase [U/ml] (IQR)	0.48 (0.26–0.88)	0.48 (0.25–0.88)	0.48 (0.28–0.9)	0.53
Preoperative lipase [U/ml] (IQR)	0.79 (0.45–1.93)	0.85 (0.31–2.38)	0.79 (0.54–1.48)	0.31
Preoperative bilirubin [mmol/l] (IQR)	12.4 (6.4–32.7)	13.3 (6.5–50.3)	11.6 (6.4–24.2)	0.02
Preoperative CRP [mg/l] (IQR)	4.8 (1.8–13.6)	5.6 (1.8–15.3)	4.4 (1.8–12)	0.58
*ASA score [n (%)]*				
1	16 (3.8)	9 (4.1)	7 (3.5)	0.08
2	166 (39.7)	74 (33.8)	92(46.2)	
3	229 (54.8)	132 (60.3)	97(48.7)	
4	7 (1.7)	4 (1.8)	3 (1.5)	
Diabetes [*n* (%)]	132 (31.5)	91 (41.6)	41 (20.5)	< 0.01
Insulin-dependent diabetes (IDDM) [*n* (%)]				
Neoadjuvant chemotherapy [*n* (%)]	41 (9.9)	25 (11.6)	16 (8)	0.23
Smoking [*n* (%)]	100 (24.4)	53 (24.5)	47 (24.2)	0.94
Drinking [*n* (%)]	88 (21.6)	51 (23.8)	37 (19.1)	0.24
Preoperative biliary drainage [*n* (%)]	175 (42.2)	99 (45.4)	76 (38.6)	0.16
*Operations performed [n (%)]*				
Partial pylorus-preserving PD (PPPD)	315 (75.2)	162 (74)	153 (76.2)	0.74
Classic PD (cPD)	104 (24.8)	57 (26)	47 (23.5)	
*Indications for resection [n (%)]*				
Chronic pancreatitis	35 (8.4)	19 (8.7)	16 (8)	< 0.01
Malignancy	350 (83.5)	192 (87.7)	158 (79)	
Benign	34 (8.1)	8 (3.7)	26 (13)	

*IQR*, interquartile range; *BMI*, body-mass-index;* POH*, postoperative hyperamylasemia;* PD*, pancreaticoduodenectomy; *CRP*, C- reactive protein

**Fig. 1:**
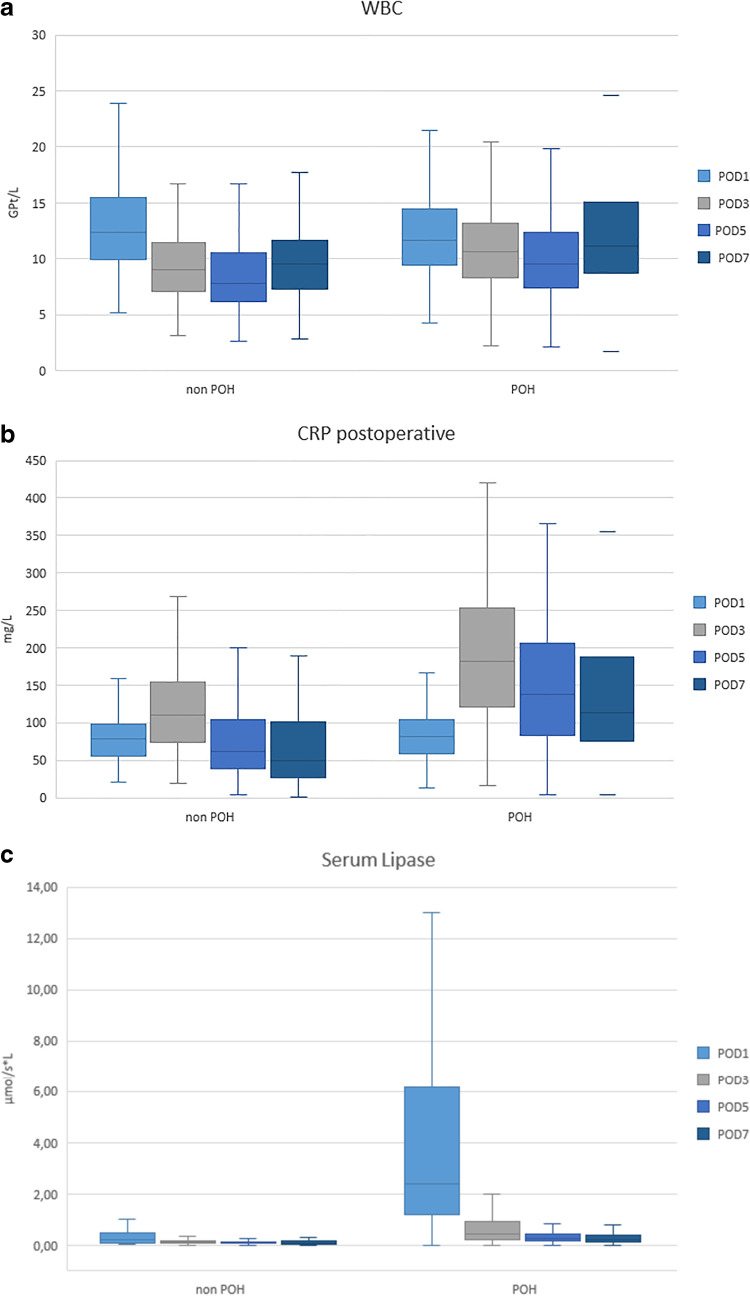
Distribution of white blood cells (WBC) (**A**); C-reactive protein (CRP) (**B**); and serum lipase (**C**) values in serum on postoperative days (PODs) 1, 3, 5, and 7 in association with and without postoperative hyperamylasemia (POH and non-POH, respectively)

POH most frequently occurred in patients with a soft pancreatic texture and small pancreatic duct diameter. Table 2 characterizes the morbidity, which was significantly higher for the POH group, with an increased rate of POPF (62% vs. 13.2%, *p* < 0.001), CR-POPF (49.5% vs. 11.4%, *p* < 0.001), abscess (38.3% vs. 15.3, *p* < 0.001), PPH (22.8% vs. 5.1%, *p* < 0.001), and CDC > 2 (52.5% vs. 25.6%, *p* < 0.001) than that found in the non-POH group [15]. Serum lipase values on PODs 1, 3, 5, and 7 were significantly higher in patients with POH than those found in the non-POH group, as shown in Fig. 1. Moreover, the CRP level and number of leukocytes, as indicators of inflammation, were significantly increased in the POH group on PODs 3, 5, and 7 compared with those in the non-POH group (Fig. 1).

**Table 2:** Peri- and postoperative analysis

	Overall	Non-POH	POH	*p*-value
Duration of operation [minutes] (IQR)	352 (295–425)	356 (293–429)	358 (298–431)	0.5
Portal vein resection [*n* (%)]	132 (31.6)	90 (41.3)	42 (21)	** < **0.001
Intraoperative blood loss [*n* (%)]	600 (400–900)	600 (400–950)	500 (400–800)	0.41
Intraoperative RBT [*n* (%)]	61 (14.6)	34 (15.5)	27 (13.6)	0.59
Pancreatic texture [*n* (%)]				
Firm	152 (47.2)	121 (73.3)	31 (19.7)	** < **0.001
Soft	170 (52.8)	44 (26.7)	126 (80.3)	
Pancreatic duct diameter [mm] (IQR)	4 (3–5)	5 (3–7)	3 (2–4)	** < **0.001
POPF [*n* (%)]	153 (36.5)	29 (13.2)	124 (62)	** < **0.001
CR-POPF [*n* (%)]	124 (29.6)	25 (11.4)	99 (49.5)	** < **0.001
Deep organ space infection [*n* (%)]	108 (26.2)	33 (15.3)	75 (38.3)	** < **0.001
PPH [*n* (%)]	56 (13.8)	11 (5.1)	45 (22.8)	** < **0.001
Major complication (CDC > 2) [*n* (%)]	161 (38.4)	56 (25.6)	105 (52.5)	** < **0.001
Rescue complete pancreatectomy [*n* (%)]	30 (7.2)	4 (1.8)	26 (13)	** < **0.001
30-day mortality [*n* (%)]	23 (5.5)	11 (5)	12 (6)	0.66
90-day mortality [*n* (%)]	30 (7.2)	13 (5.9)	17 (8.5)	0.31

*RBT*, rapid blood transfusion;* IQR*, interquartile range;* POH*, postoperative hyperamylasemia;* POPF*, postoperative pancreatic fistula;* CR-POPF*, clinically relevant POPF;* CP*, complete pancreatectomy; *PPH*, postoperative hemorrhage;* CDC*, Clavien-Dindo classification

If a fluid collection was detected in a postoperative CT scan as examination for inflammation focus, a CT-assisted drainage was placed. The median postoperative period for interventional drainage in patients with complications was 10 (IQR 4–27) days.

Rescue pancreatectomy was performed more frequently during the hospital stay in patients with POH than in those without, and the difference was significant (13% vs. 1.8%, *p* < 0.001). Although there was no significant difference in the 30- and 90-day mortality between the groups, those in the POH group were slightly increased.

### Analysis of risk factors for POPF

**Fig. 2:**
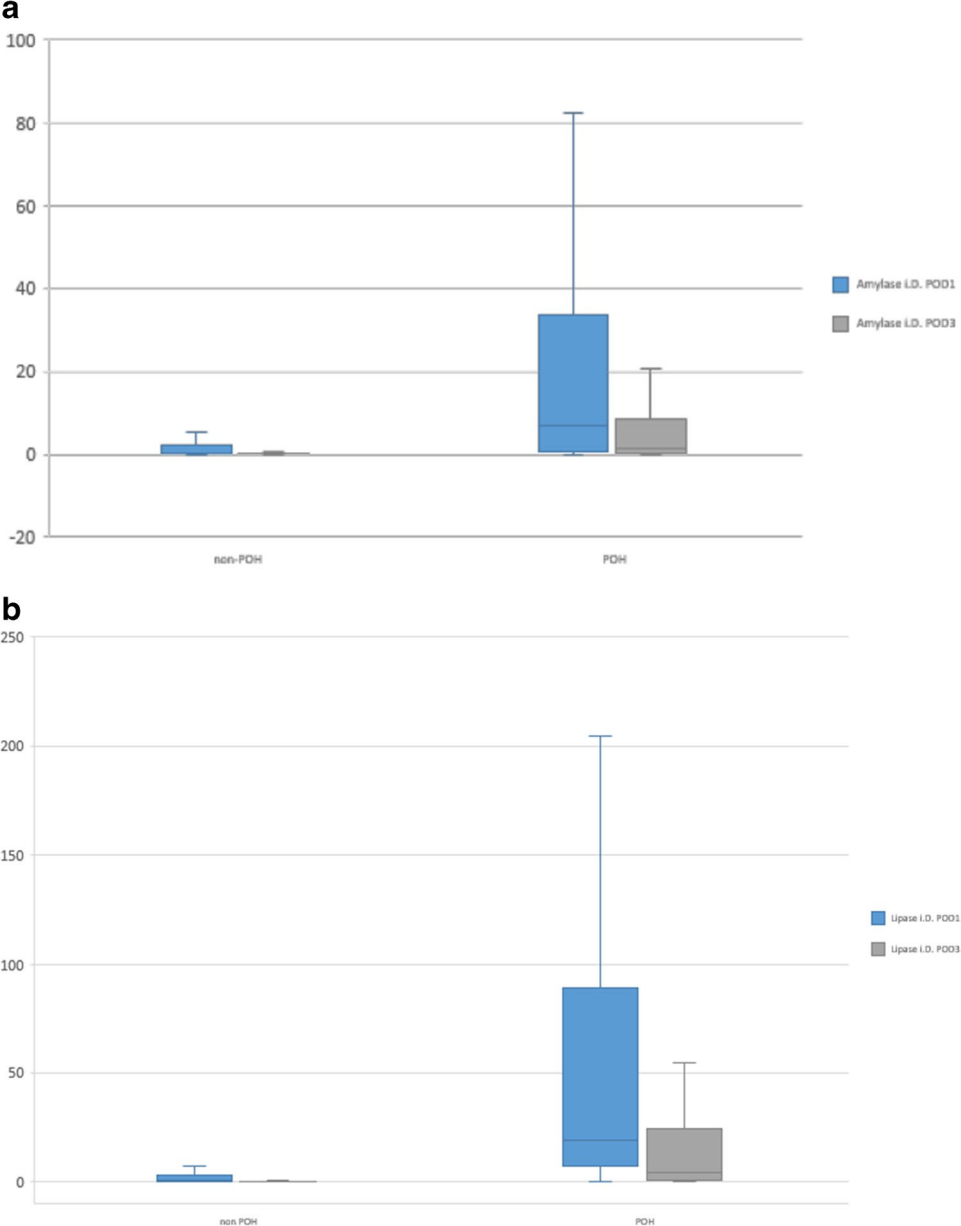
Drainage amylase and lipase levels on postoperative day 1 in association with postoperative hyperamylasemia (POH and non-POH, respectively)

Amylase levels in the drainage fluid on POD 1 differed considerably between the groups; however, the difference was not statistically significant. In contrast, the lipase levels were significantly higher in association with POH on POD1 and POD3 (Fig. 2). The importance of amylase and lipase levels for predicting POPF has been previously reported in another study [21]. We observed in contrast to POPF a significant increase of the lipase levels in association with POH.

In the univariate analysis, portal vein resection, pancreatic duct diameter, soft pancreatic texture, POH, POHL on POD 1, and CRP on POD 3 were identified as possible predictors of POPF, while in the multivariate analysis, BMI, pancreatic texture, and POH were found to be independent predictors for POPF (Tables 3 and 4).

**Table 3:** Uni- and multivariate analysis of predictors of POPF after pancreatic resection

	Univariate analysis			Multivariate analysis		
Parameters	OR	95% CI	*p*-value	OR	95% CI	*p*-value
Age	1.002	0.985–1.020	0.8	n.a		
BMI	1.110	1.060–1.162	** < **0.001	1.161	1.058–1.274	** < **0.01
Diabetes	0.492	0.315–0.77	** < **0.01	0.635	0.291–1.385	0.25
Chronic pancreatitis	0.359	0.181–0.713	** < **0.01	1.635	0.318–8.413	0.56
Nicotine	0.995	0.625–1.583	0.98	n.a		
Alcohol	0.881	0.539–1.439	0.61	n.a		
Preoperative biliary stent	0.914	0.613–1.363	0.66	n.a		
Neoadjuvant chemotherapy	0.521	0.249–1.089	0.08	0.46	0.135–1.572	0.22
*Diagnosis*						
	Reference			Reference		
Malignant disease	1.827	0.811–4.118	0.15	1.815	0.276–11.921	0.54
Benign disease	9.425	3.233–27.473	** < **0.001	5.699	0.642–50.545	0.12
Portal vein resection	0.358	0.224–0.573	** < **0.001	0.61	0.268–1.391	0.24
Soft pancreatic texture	12.258	7.050–21.314	** < **0.001	3.860	1.705–8.742	** < **0.01
Pancreatic duct diameter	0.699	0.608–0.804	** < **0.001	0.997	0.837–1.186	0.97
RBT transfusion intraoperative	0.817	0.461–1.445	0.49	n.a		
Postoperative amylasemia day 1	10.690	6.589–17.344	** < **0.001	8.217	3.558–18.979	** < **0.001

*POPF*, postoperative pancreatic fistula;* BMI*, body mass index;* CI*, confidence interval; *OR*, odds ratio; *RBT*, rapid blood transfusion;* n.a*., not available

**Table 4:** Uni- and multivariate analysis of predictors of rescue complete pancreatectomy after PD

	Univariate analysis			Multivariate analysis		
Parameters	OR	95% CI	*p*-value	OR	95% CI	*p*-value
Age	1.010	0.976–1.044	0.58	n.a		
BMI	1.064	0.987–1.147	0.1	1.105	0.983–1.243	0.09
Diabetes	0.297	0.102–0.867	0.03	0.265	0.259–4.025	0.1
Chronic pancreatitis	0.414	0.096–1.781	0.24		n.a	
Nicotine	0.852	0.336–2.162	0.74	n.a		
Alcohol	1.729	0.759–3.938	0.19	0.589	0.178–1.955	0.39
Preoperative biliary stent	0.766	0.355–1.652	0.66	n.a		
Neoadjuvant chemotherapy	0.606	0.139–2.631	0.5	n.a		
*Diagnosis*						
	Reference			Reference		
Malignant disease	1.346	0.307–5.912	0.69	n.a		
Benign disease	1.591	0.250–10.131	0.69	n.a		
Portal vein resection	0.610	0.256–1.452	0.61	n.a		
Soft pancreatic texture	4	1.464–10.925	** < **0.01	1.022	0.259–4.025	0.98
Pancreatic duct diameter	0.612	0.441–0.849	** < **0.01	0.766	0.497–1.181	0.23
RBT transfusion intraoperative	1.123	0.913–1.381	0.27	0.826	0.419–1.630	0.58
Postoperative amylasemia day 1	8.032	2.751–23.450	** < **0.001	6.383	1.164–35.014	0.03
Postoperative lipasemia day 1	4.926	0.618–39.290	0.13		n.a	
Postoperative CRP day 1	0.715	0.341–1.498	0.37	0.857	0.309–2.380	0.77
Postoperative LC day 1	0.807	0.388–1.682	0.57	0.905	0.323–2.541	0.85

*BMI*, body mass index;* CI*, confidence interval;* OR*, odds ratio;* RBT*, rapid blood transfusion; *CRP*, C-reactive protein;* LC*, leukocytosis; *n.a.*, not applicable

### Analysis of risk factors for rescue CP

In total, during the whole study period, we performed 31 CPs. In 25 (81%) cases, on pathologically testing the anastomosis area and pancreatic remnants, a diagnosis of necrotizing pancreatitis was reached. In the remaining six (19%) cases, the pancreatic remnants were provided for islet cell transplantation, which was carried out at the end of the surgery. Rescue CP was always performed in cases with a severe septic postoperative course and life-threatening complications at a wide variable interval to the primary surgery in median 9.5 (IQR: 6.25–12). Mortality after CP was high (*n* = 11; 35.4%). The 30- and 90 days mortality in the CP group were (*n* = 6; 19.4%) and (*n* = 5; 16.1%) respectively.

In the multivariate analysis, POH was independently associated with rescue CP (odds ratio [OR] 6.383, 95% confidence interval [CI] 1.164–35.014, *p* = 0.03).

### Analysis of POH, leukocytosis, and CRP

Since other biochemical changes on POD1 were related to postoperative morbidity in patients with POH, we analyzed their effect on patient outcome in association with POH (Table 5). Patients with a normal serum amylase level were compared to patients with POH alone; to patients with POH, leukocytosis, or CRP elevation; or to patients with combination of all three conditions. There were significantly more cases with postoperative complications, such as CDC > 2 (55.6%), abscess (43.2%), CR-POPF (51.6%), and PPH (21.6%), when all three risk factors were present. Furthermore, almost half of the patients who underwent rescue CP (*n* = 13) showed elevations in all three parameters (*p* < 0.001), while none of the patients with POH alone underwent rescue CP. The hospital stay was significantly prolonged in patients with POH or with elevations in the number of white blood cells (WBC) or CRP level.

**Table 5:** Postoperative morbidity stratified by POH occurrence alone on POD1, or with leukocytosis, CRP, or both parameters

	Non-POH (*n* = 153)	POH alone (*n* = 5)	POH and leukocytosis (*n* = 17)	POH and CRP (*n* = 182)	POH, CRP, and leukocytosis (*n* = 249)	*p*-value
CDC > 2 [*n* (%)]	56 (25.6)	2 (22.2)	5 (33.3)	28 (56)	70 (55.6)	** < **0.001
CR-POPF [*n* (%)]	25 (11.4)	0	5 (33.3)	29 (58)	65 (51.6)	** < **0.001
PPH [*n* (%)]	11 (5.1)	0	1 (6.7)	17 (35.4)	27 (21.6)	** < **0.001
Abscesses [*n* (%)]	33 (15.3)	2 (22.2)	4 (28.6)	15 (31.3)	54 (43.2)	** < **0.001
CP [n (%)]	4 (1.8)	0	2 (13.3)	11 (22)	13 (10.3)	** < **0.001
Hospital stay [days] (IQR)	14 (11—22)	15 (11.5–22)	15 (10–24)	25.5 (12–49)	21 (14–34)	** < **0.001
30-day mortality [*n* (%)]	11 (5)	0	0	3 (6)	9 (7.1)	0.7
90-day mortality [*n* (%)]	13 (5.9)	1 (11.1)	0	5 (10)	11 (8.7)	0.57

*CRP*, C-reactive protein;* POH*, postoperative hyperamylasemia; *IQR*, interquartile range;* CR-POPF*, clinically relevant postoperative pancreatic fistula;* CP*, complete pancreatectomy; *PPH*, postoperative hemorrhage; *CDC*, Clavien-Dindo classification

## Discussion

In 2016, acute pancreatitis after pancreatectomy was reported as a new entity in postoperative complications and was defined by summarizing the biochemical evidence of pancreatic inflammation [12]. Postoperative morbidity was found to be significantly increased in patients with POH, making serum amylase an important indicator of the postoperative course. Thus, POH after pancreatectomy has received increasing attention [9–11, 22, 23]. In the absence of a general definition for PPAP, the overall reports were based on widely differing and divergent criteria. PPAP was often defined based on the revised Atlanta classification for acute pancreatitis or by an elevation in postoperative serum amylase above the upper limit [9].

In our retrospective study, we analyzed the outcomes of two patient groups––those with and without POH––and identified significant higher frequencies of postoperative morbidity (CDC > 2), POPF, PPH, intraabdominal abscess, and CP when POH occurred. In concordance with other reports, our data supports a strong proportional association between POH on POD 1 and increased morbidity [1, 3, 4, 6, 8]. These findings suggest that POH may be a biochemical marker of concern.

Moreover, POH was found to significantly correlate with the risk factors for POPF, such as soft pancreatic texture and small pancreatic duct diameter. It was also shown to be an independent predictor for POPF in the multivariate analysis. Considering that mortality after POPF grade C remains very high (at > 55%), POH should also be regarded as an early and readily assessable indicator of considerable importance for the development of septic postoperative complications and should be taken into account to mitigate morbidity at the early postoperative stage [4, 24].

Nevertheless, the exact mechanism for the development of POH and its relation to POPF is still unknown and poorly studied.

In order to avoid this, suture material used should preferably be thin and knots should cause less compression to minimize tissue damage [25].

As a matter of fact, the incidence of POPF and overall morbidity were more frequent when postoperative pancreatitis was present, and these findings are supported by several other studies [19, 26]. However, a uniform definition for the latter failed in the past.

Previous research has focused on other biochemical markers, such as trypsinogen, procathepsin B, and IL-6, as indicators of inflammation in the pancreatic remnants [9, 27, 28]. Biochemical changes in the serum and drainage fluid were further analyzed to identify patients suitable for cost-effective early drainage removal and early discharge [27, 28]. Overall, biochemical markers were estimated to have a useful and important predictive impact on the postoperative course; however, the related data is scarce and diverse.

For a more comprehensive assessment of the early predictive biomarkers for inflammatory changes in the pancreatic remnants, we compared the serum lipase, CRP, and WBC dynamics in the POH and non-POH groups and found a significant increase of all three parameters if POH was present. Furthermore, complications (CDC > 2, POPF, PPH, intra-abdominal abscess, CP, and length of hospital stay) for patients with POH occurred frequently when WBC and CRP levels were elevated. These findings might indicate a more exponentiated predictive value of POH when other inflammatory parameters in the serum are also elevated.

To achieve a better understanding of the biochemical dynamics and possible predictors for pancreatitis, we focused on patients who required rescue CP for a severe septic condition after pancreatectomy 4.

Similar to the results for POPF, risk factors like pancreatic texture and diameter and POH correlated significantly to the need for CP. Moreover, POH was found to be an independent predictor for CP.

When the pancreatic remnants were histologically tested after the salvage procedure, acute/necrotizing pancreatitis in 81% of the examined specimens was found. We can consequently speculate that POH, when other risk factors are also present, might be considered as a very early predictor for severe inflammatory changes in the pancreatic remnants.

However, in line with several study findings, we observed POH on POD1 in half (51.0%) of the patients after PD [9, 10]; however, not all of them suffered from complications in their postoperative course. For this reason, several authors doubt that elevated serum amylase alone leads to PPAP [9, 10]. Our data could possibly support these results and demonstrate that elevated pancreatic enzymes are often associated with postoperative biochemical changes when complications occur. Notably, no cases of CP were performed when only POH was present.

The ISGPS’ definition and grading system for PPAP enables a systematic study of its incidence to provide a better understanding of its impact on and association to the risk factors [13]. An analog to the biochemical leakage in POPF grading, POH alone was defined as postoperative hyperamylasemia with no clinically relevant impact, and postoperative pancreatitis was only considered if clinical events with increased morbidity occurred with a radiological evidence of acute pancreatitis.

Our findings concur with those of previous literature reporting that POH alone was not a strong indicator for PPAP [10]. This finding is in line with the new definition of ISGPS [13]. Still, its predictive value for postoperative morbidity is evident and is significantly enhanced if other inflammatory parameters are elevated. According to our findings and considering the new definition of PPAP, the additional biochemical indicators are essential to predict a severe course in case of POH after PD. Therefore, it is important to investigate the association of POH with other predictors in the development of PPAP from the outset and to perform a CP, if necessary, at an early stage.

Furthermore, the radiological findings related to PPAP should be better examined and understood, as peripancreatic fluid may be present postoperatively, and pancreatic necrosis may be identified after a few days, making it a later indicator for PPAP [29].

Although this study provides important information related to the new definition of PPAP, it clearly has some limitations. The most important one is its retrospective character. There is an increased risk for recall bias especially concerning patient data and operation variables. As we present data from a single center, a generalization of our results is limited. In addition, the heterogeneity of our patients (benign and malignant entities) contributes to a limitation in generalizing a statement. After a review of our results, a profit in patient care must be expected from standardized postoperative blood tests. It however remains unknown if early detection of high amylase levels in serum leads to an improved clinical management for preventing complications and improving outcome. Furthermore, only 31 patients required CP, which is insufficient for a representative statistical result. A potential limitation of the statistical analysis is also the multivariate stepwise regression model, which may be associated with bias in the parameter estimation. However, we believe that in the current analysis, our study has included the important (central) variables needed for the relationship to POPF and CP and shows the significant difference between the two groups of patients studied.

Nevertheless, we investigated few important issues that need to be addressed for the diagnosis of PPAP and that may predict the severity of complications. We showed that POH is an important indicator for morbidity, and in combination with other biochemical changes, it is often associated with more severe complications in the postoperative period.

## Conclusion

Now that a universal definition for PPAP is available, future studies should investigate the specific pathophysiology of this entity. Moreover, it will be essential to implement readily assessable biochemical and clinical predictors for the early postoperative detection of PPAP and the associated complications and to consider a complementary pancreatectomy before septic complications occur. Finally, the relationship between POPF and PPAP should be analyzed in future studies to determine whether these are different definitions of the same postoperative condition.

### Supplementary Information

Below is the link to the electronic supplementary material.

Supplementary file1 (DOCX 30 KB)

## Data Availability

Data is available on request from the corresponding author.
